# Health professionals’ perception of pharmaceuticals procurement performance in public health facilities in Southwestern Ethiopia

**DOI:** 10.1186/s40545-021-00344-5

**Published:** 2021-07-09

**Authors:** Gotuma Negera, Hailu Merga, Tadesse Gudeta

**Affiliations:** 1 Jimma Medical Center, Jimma, Ethiopia; 2 Department of Epidemiology, Institute of Health, Jimma University, Jimma, Ethiopia; 3 School of Pharmacy, Institute of Health, Jimma University, Jimma, Ethiopia

**Keywords:** Health professionals’ perception, Pharmaceutical procurement performance, Public health facilities, Jimma

## Abstract

**Background:**

Pharmaceutical’s procurement is a core component of logistics management, and has a significant influence on product availability, and total supply chain costs. In Ethiopia, there are few studies on this topic where almost all of them were from suppliers’ perspectives and entirely quantitative. This study, therefore, aimed to assess health professionals’ perceptions about pharmaceuticals procurement performance in public health facilities in southwest Ethiopia.

**Methods:**

A facility-based cross-sectional study complemented with a qualitative method was conducted from March 20 and April 30, 2019. We collected the quantitative data through self-administered structured questionnaires from pharmacy staff and document review using checklists. EpiData version 3.1 and SPSS version 20 were used for data entry and analysis, respectively. Descriptive statistics were done for quantitative data. Qualitative data were gathered through face-to-face in-depth interviews and analyzed using thematic analysis technique.

**Results:**

Regarding respondents’ perception of accountability in pharmaceutical procurement, 110 (57.9%) agreed or strongly agreed that their facilities adopt and use standard treatment guidelines and facility-specific medicine lists. Concerning competitiveness, 139 (62.6%) of the participants either disagreed or strongly disagreed that their facilities used formal suppliers’ qualifications based on service reliability and financial capacity. Regarding efficiency, 146 (76.8%) disagreed or strongly disagreed that their facilities develop a mechanism for prompt, reliable payment to lower medicine prices. The qualitative analysis identified staff workforce and competency, budget shortages, suppliers’ uncertainty, and pharmaceutical non-availability as challenges for procurement management performance.

**Conclusion:**

The results indicated that participants perceived procurement performance of their facilities as poor. Therefore, staff development, fundraising options, monitoring and evaluation, coordination and collaboration can improve procurement practice and performance.

## Background

Pharmaceutical’s procurement is a core component of logistics management, and has a significant influence on availability, and total supply chain (SC) costs [1]. In most developing countries, sales of these goods represent the single highest spending on health care after personnel expenditure and also take a significant share of foreign currencies [2]. As a result, public procurement activities need to work towards the fulfillment of objectives, such as the acquisition of high-grade products in a sufficient amount for the correct cost and designated time under the contractual means using government budget [3–5]. To meet the intended objectives, every effective procurement activity must follow the fundamental concepts of transparency and employ open bidding unless there are valid grounds for purchasing from a single vendor. The procurement procedures should underline the fair treatment of all bidders regardless of race, ethnicity, or political affiliation [5, 6]. Moreover, rational pharmaceuticals procurement practices dictate that purchases should be limited to the essential drug list, based on generic names, in large-volumes whenever possible, in quantities based on a reasonable estimation of real requirements, consistency, quality control, and supplier certifications [7].

A previous study revealed that the procurement malpractices in the form of bribes have resulted in the depletion of government funds through the inappropriate allocation of budget and quality defective goods, services, or works [8]. In some countries, the use of paper-based systems is reported to encourage greater incentives for fraudulent practices [9]. Additionally, while various national procurement structures exist across developing countries, the supply of vital drugs to many of these communities relies heavily on public funds, foreign funding mechanisms, and donor agencies. For example, the Global Fund spent $ 32.6 billion in 2017 to support HIV/AIDS, tuberculosis, malaria services, and health supplies, which represents almost 40% of grant expenses [10]. However, the public agencies of these countries lack the technical resources to carry out the procurement process strategically. Consequently, insufficient preparation and analysis and the use of ineffective procurement processes lead to significant stock out of medicines and wastage of resources. This, in turn, forces patients to seek medicines from other private outlets where prices are much higher and likely to be of dubious quality [11, 12].

Ethiopia is one of the countries with a high annual demand for pharmaceuticals, up to USD 1 billion [13]. Under normal circumstances, the Ethiopian Pharmaceutical Supply Agency (EPSA) is mandated to fulfill these demands [14]. Nonetheless, as the agency aggregates requirements for health facilities and conducts bulk procurement, it may sometimes take longer lead times [15]. Hence, to avoid service interruptions, the health facilities may purchase the required pharmaceuticals from private wholesales after receiving a letter attesting to the stock out of the products at the agency hubs [16]. Studies show that the majority of public health facilities in Ethiopia face a frequent shortage of medicines [17–21] and the percentage availability does not exceed 80% [22]. This is contrary to the aim of the Sustainable Development Goal (SDG), which promotes "access to safe, effective, high quality, and affordable essential medicines and vaccines for all" as a core element of Universal Health Coverage [23]. Inadequate access to pharmaceuticals significantly affects quality of health service delivery [24, 25]. As a result, people suffer from a low to a higher level of health issues, from no relief to the immense pain of a child’s ear to women’s death during delivery with profuse bleeding and patients dying from preventable and curable diseases [26].

Efforts must, therefore, be made to improve the availability and affordability of essential medicines, one of which is efficient procurement practices [27]. Cost-effective procurement processes can vastly improve efficiency, access to vital drugs, and service performance [11]. Studies conducted in Ethiopia in the area of pharmaceuticals supply chain management focus basically on accessibility, inventory management, medicines price, affordability, pharmaceuticals waste practice, and information systems [17, 21, 22, 25, 28–30]. Although procurement management is one of the primary logistics practices and accounted for 40% of the health care expenditure [2], studies on this issue and health professionals’ perception about the issue are frequently overlooked. The available studies are from a supplier perspective and entirely quantitative [31, 32]. Moreover, there is a lack of study on health professionals’ perception about how health facilities use resources (efficiency), the extent to which they follow transparent procedures and the level of accountability, and whether competitiveness prevails in the procurement process, from supplier selection to the award of contracts. The aim of this study was, therefore, to assess the perception of health professionals’ on the performance of pharmaceutical procurement in public health facilities in Jimma zone, Southwestern Ethiopia.

## Methods

### Study settings and period

A facility-based cross-sectional study supplemented with a qualitative method was conducted in public health facilities of Jimma zone from March 20 to April 30, 2019. Jimma zone is one of the zones in Ethiopia’s Oromia regional state and located 350 km away from the capital Addis Ababa to the southwest part of the country. The zone has 19 administrative districts of which the Jimma city is the capital. According to the projection of the Central Statistical agency, the total population of Jimma zone was estimated to be 3,729,232 in 2019. So far, there are 778 health institutions in the zone, including seven public hospitals, 122 health centers, 486 health posts, 156 private clinics, four non-governmental organization clinics, two military hospitals, and one private hospital.

### Population and sampling

The source populations were all health professionals’ and health institutions providing service to the community in Jimma Zone. The study population included all pharmacy staff (including health care professionals working as pharmacy staff) in government hospitals and health centers operating for at least 1 year. The respondents were selected from public hospitals and health centers since these facilities are dedicated to providing services to large segments of the population and are funded directly by the federal and regional governments. The reason that health posts were excluded was that they serve as dispensing units in the health system of Ethiopia and are supplied directly by health centers. As private health care facilities are profit-making entities, their supply chain activities were assumed to be predominantly managed by owners and issues with purchasing practices and performance may not be serious concerns.

Concerning sampling procedure, though the logistics indicators assessment tool (LIAT) recommends 15% of the health facilities [33], to get a large sample size, all pharmacy staffs in the hospitals (seven) were considered as those hospitals found in different woredas and had provided service for more than a year. Besides, hospitals are expected to do frequent purchasing of pharmaceuticals since more customers seek health services. To select the participants in health centers, districts were classified based on the number of health center/s they have. Then, we used a lottery method to select 12 of the 19 districts but deliberately included Jimma City because it acts as the capital and have more residents. Subsequently, Agaro, Dedo, Gera, Goma, Gumay, Kersa, Limmu Kossa, Manna, Omo Nada, Seka Chekorsa, Setema, and Shebe Sombo, including Jimma city, were visited. We selected 30% of the total health centers (*n* = 37) proportionally in those nominated districts and Jimma city using a simple random sampling technique. According to zonal health department data, there were 220 pharmacy staffs in these selected health facilities. But we included those dealing with drug purchases based on information provided by heads of pharmacy departments and managers of health centers. Accordingly, we distributed the questionnaires to 195 participants. A total of 14 key informants participated in the qualitative study, including department heads, executives or medical directors of hospitals, health center directors, and procurement (finance) department coordinators. The sample size depended on the information saturation and the interview ceased when the same information was reiterated by subsequent interviewees. We also reviewed some procurement documents of the last year, July 8, 2017, to July 7, 2018 (corresponding with the Ethiopian fiscal year), to compliment the information obtained from health professionals. The documents included, purchase invoice and Model-19. Model-19 is a logistical transaction document used by health facilities to receive pharmaceuticals from designated suppliers. Accordingly, a total of 626 purchase invoices (232 from hospitals and 394 from health centers) and 36 Model-19 pads (17 in health centers and 19 in hospitals) were reviewed. In document review, all donations and procurement made by other stakeholders were excluded because they were not performed by the health institutions under the study.

### Data collection tool and procedure

The quantitative data were collected by pharmacy professionals using self-administered structured questionnaire and checklists covering all the aspects of the study variables. The self-administered questionnaire had two parts. The first part dealt with socio-demographic characteristics. The second section contained the participants’ perception of procurement performance. The questions on the second part were constructed based on a 5-point ‘Likert’s scale response continuum (i.e., strongly agree = 5, agree = 4, neutral = 3, disagree = 2, and strongly disagree = 1). The questions were developed on the basis of good procurement practice measures, including accountability, transparency, competitiveness, and efficiency. Level of accountability was assessed through sub-indicators including, availability of Essential Medicine Lists (EML) to guide procurement, development of specification, quantification based on consumption data, keeping procurement records, supplier’s evaluation, and procurement performance evaluation. Transparency was measured through assessing if the procurement unit develops and follow written procedures, no separate deals with non-contracted supplier, the unit makes the procurement process, and results accessible to public and presence of functional Drugs Therapeutic Committee (DTC). Statements used to measure the level of competitiveness included: the facility uses generic names, uses competitive method, supplier post-qualification, evaluation and award of the supplier based on cost, service reliability and financial capability in tenders. Level of efficiency or value for money was assessed using questions like if the facilities select cost-effective medicines, purchases on limited list to increase quantities and reduce price, notifies separated deliveries, executes prompt payment, and conducts an annual financial audit. Both the checklists and the questionnaires were adapted and customized from methodology assessment for the procurement system (MAPS), international handbook of public procurement, and managing access to medicines and health technologies [2, 34, 35].

Purchase invoices for all procurement methods (open tender, request for quotation, direct procurement) from private suppliers, and EPSA were reviewed. DTC terms of reference and minutes, availability of updated facility EML were also assessed using a checklist. Data collectors and supervisors were trained for 2 days. The collected data were checked for consistency and completeness each day at the end of data collection. For the qualitative data collection, key informant interviews (KII) were conducted with pharmacy heads, department heads, finance, PHCU (Primary Health Care Unit) directors and medical director or hospital CEO. The key informants with a different profession and position were purposively selected to get heterogeneous responses. Data were collected using a semi-structured interview guide containing open-ended questions. The interviews were conducted by the principal investigator to maintain consistency throughout the interview. The interviewer arranged schedule with the key informants to identify convenient time and place. Interviews were conducted by recording with digital audio recorder using critical incident techniques that allow participants to tell the story (what?) that generate details by posing probing ‘when’, ‘where’ ‘why’ and ‘how’ questions. Data collection was conducted in the regional language, Afan Oromo and the interview took 20–30 min. The interviews were terminated upon a saturation of responses on each question.

### Data analysis

The STrengthening the Reporting of OBservational studies in Epidemiology (STROBE) checklist was used to analyze and report data [36]. The collected quantitative data were sorted and entered on EpiData version 3.1 and analyzed using Statistical Package for Social Sciences (IBM SPSS Statistics for Macintosh, Version 20.0. Armonk, NY). Then, descriptive statistical analyses were computed. The results were presented in the forms of tables and charts. Qualitative data were analyzed using thematic analysis technique. Accordingly, the record was directly translated and transcribed into English language. Themes were deduced manually from the transcribed data guided by the specific objectives. Finally, the data were triangulated with quantitative findings during discussions.

## Results

### Findings of the quantitative assessment

#### Socio-demographic characteristics

One hundred ninety-five participants involved in the study of which 190 of them responded properly, with a response rate of 97.44%. Among those respondents, about half, 94 (49.5%), were from hospitals and 143 (75.3%), of them were males. One hundred thirty-three (70%) were pharmacy professionals and about half, 91 (47.9%), of them had less than 2 years of experience at the health facilities. More than three-fourths, 148 (77.9%), of the study participants were involved in pharmaceutical procurement directly (Table 1).

**Table 1:** Socio-demographic characteristics of the health professionals in public health facilities of Jimma zone, southwest Ethiopia, April, 2019 (*n* = 190)

Variables	Characteristics	Frequency (%)
Health facility level	Hospitals [7]	94 (49.5)
	Health centers [37]	96 (50.5)
Gender	Male	143 (75.3)
	Female	47 (24.7)
Educational level	Diploma	56 (29.5)
	First degree	133 (70)
	Master’s degree	1 (0.5)
Type of profession	Pharmacy	133 (70)
	Clinical nurse	54 (28.4)
	Health officer	2 (1.1)
	Midwifery	1 (0.5)
Service year in that facility	Less than 2 years	91 (47.9)
	2–5 years	66 (34.7)
	Greater than 5 years	33 (17.4)
Manages pharmaceutical procurement	Directly	148 (77.9)
	Indirectly	42 (22.1)

#### Health professionals’ perception on public pharmaceutical procurement performance

The health professionals’ pharmaceutical procurement performance was assessed based on the principles of good procurement practices including accountability, transparency, competitiveness, and efficiency.

#### Perceptions on level of accountability

Respondents were asked to consider seven statements/questions regarding accountability in public health facilities pharmaceutical procurement. Ninety-one (47.9%) of them agreed or strongly agreed that the facility adopts and uses standard treatment guidelines (STGs) and facility-specific medicine lists (Table 2).

**Table 2:** Health professionals’ perception on level of accountability pharmaceutical procurement in public health facilities of Jimma Zone, April 2019 (*n* = 190)

Accountability	SD*	D*	N*	A*	SA*
	*n* (%)	*n* (%)	*n* (%)	*n* (%)	*n* (%)
My facility adopts and uses STGs and facility-specific medicine list	11 (5.8)	61 (32.1)	8 (4.2)	91 (47.9)	19 (10.0)
Facility’s procurement present results to the appropriate public supervisory	13 (6.8)	49 (25.8)	14 (7.4)	103 (54.2)	11 (5.8)
Due to lack of reliable specifications no unfit products procured	12 (6.3)	47 (24.7)	26 (13.7)	76 (40.0)	29 (15.3)
My facility reliably quantifies consumptions	22 (11.6)	111 (58.4)	9 (4.7)	41 (21.6)	7 (3.7)
My facility uses key indicators such as ratio of prices to world market and supplier lead time to evaluate supplier performance	58 (30.5)	90 (47.4)	11 (5.8)	28 (14.7)	3 (1.6)
Reports key procurement performance indicators against target plans	70 (36.8)	77 (40.5)	13 (6.8)	22 (11.6)	8 (4.20
Uses a formal monitoring system to ensure continued supplier qualifications	27 (14.2)	63 (33.2)	8 (4.2)	80 (42.1)	12 (6.3)

SD* strongly disagree, D* disagree, N* neutral, A* agree, SA* strongly agree

#### Perceptions on level of transparency

With regard to transparency, respondents were asked to consider five questions to indicate their level of agreement. Eighty-two (43.2%) disagreed or strongly disagreed that different committees, units and individuals involved in selection, quantification or award (Table 3).

**Table 3:** Health professionals’ perception on level of transparency of pharmaceutical procurement in public health facilities of Jimma zone, April 2019 (*n* = 190)

Transparency	SD*	D*	N*	A*	SA*
	*n* (%)	*n* (%)	*n* (%)	*n* (%)	*n* (%)
My facility develops and follows written procedures for all procurement actions	28 (14.7)	54 (28.4)	14 (7.4)	87 (45.8)	7 (3.7)
Makes the tender process and results information public to the maximum extent	37 (19.5)	102 (53.7)	6 (3.2)	41 (21.6)	4 (2.1)
There are no separate deals with non-contracted suppliers	10 (5.3)	39 (20.5)	41 (21.6)	89 (46.8)	11 (5.8)
Different committees, units or individuals involved selection, quantification, approval and award of contracts	32 (16.8)	82 (43.2)	6 (3.2)	64 (33.7)	6 (3.2)
The procurement system in this facility is operating in a totally transparent manner	14 (8.4)	53 (27.9)	43 (22.6)	74 (38.9)	6 (3.2)

SD* strongly disagree, D* disagree, N* neutral, A* agree, SA* strongly agree

#### Perceptions on level of competitiveness

Regarding competitiveness, 78 (41.1%) of the participants either disagreed or strongly disagreed with the health facilities selection of suppliers on the basis of service reliability, financial capacity. Besides, 87(45.8%) of them disagreed or strongly disagreed that health facility uses competitive bidding in all except emergency purchases (Table 4).

**Table 4:** Health professionals’ perception on level of competitiveness pharmaceutical procurement in public health facilities of Jimma zone, April 2019 (*n* = 190)

Competitiveness	SD*	D*	N*	A*	SA*
	*n* (%)	*n* (%)	*n* (%)	*n* (%)	*n* (%)
My facility uses competitive bidding in all but very small or emergency procurement	14 (7.4)	87 (45.8)	8 (4.2)	69 (36.3)	12 (6.3)
Allows only pre-qualified suppliers to compete in restrictive tenders	15 (7.9)	55 (28.9)	8 (4.2)	104 (54.7)	8 (4.2)
Evaluates suppliers after submission of bids in open tenders	9 (4.7)	44 (23.2)	10 (5.3)	113 (59.5)	14 (7.4)
Use formal supplier qualification based on pharmaceutical quality, service reliability, cost	41 (21.6)	78 (41.1)	12 (6.3)	54 (28.4)	5 (2.6)
Use generic names	8 (4.2)	27 (14.2	13 (6.8)	117 (61.8)	25 (13.2)

SD* strongly disagree, D* disagree, N* neutral, A* agree, SA* strongly agree, M* mean

#### Perceptions on level of efficiency

Regarding efficiency in pharmaceutical procurement management, 96 (50.5%) of the participants disagreed or strongly disagreed that the health facility develops a mechanism for prompt reliable payment to lower the price. Similarly, 94 (49.5%) of them either disagreed or strongly disagreed that the procurement team notifies divided deliveries (Table 5).

**Table 5:** Health professionals’ perception on efficiency in pharmaceuticals procurement of public health facilities of Jimma zone, April, 2019 (*n* = 190)

Efficiency	SD*	D*	N*	A*	SA*
	*n* (%)	*n* (%)	*n* (%)	*n* (%)	*n* (%)
Facility procurement selects safe, effective, cost-effective medicines	8 (4.2)	46 (24.2)	10 (5.3)	114 (60)	12 (6.3)
Concentrates purchases on limited list to increase quantities and reduce price	30 (15.8)	67 (35.3)	9 (4.7)	73 (38.4)	11 (5.8)
Develops mechanisms for prompt, reliable payment that brings down prices more than bulk discounts	50 (26.3)	96 (50.5)	9 (4.7)	33 (17.4)	2 (1.1)
Conducts annual financial audit to assess compliance with procedures, payment promptness of and related factors	15 (7.9)	65 (34.2)	14 (7.4)	90 (47.4)	6 (3.2)
The facility notifies deliveries	24 (12.6)	94 (49.5)	21 (11.1	47 (24.7)	4 (2.1)

SD* strongly disagree, D* disagree, N* neutral, A* agree, SA* strongly agree

### Findings of procurement document review

The study assessed the procurement management process and reviewed purchase documents of the 2018/2019 from the selected health facilities. Four (57.1%) of the hospitals had their own EML but 20 (54%) among the health centers. With regard to DTC, five (71%) and 27 (73%) of the hospitals and health centers, respectively, had functional DTCs. Moreover, six (85.7%) hospitals and two (5.4%) HCs had procurement/tender committee. Only JUMC used a competitive tender method. Twenty-one (47.73%) of the facilities purchased through a direct method at least up to four times during the 2018/19 fiscal year (Table 6).

**Table 6:** Procurement-related factors and frequency of emergency purchase among public health facilities in Jimma Zone

	Category	*n* (%)
Availability of DTC	Hospital	5 (71.4)
	Health center	27 (73)
Procurement committee	Hospital	6 (85.7)
	Health center	2 (5.4)
Facility EML	Hospital	4 (57.1)
	Health center	20 (54)
Frequency of direct-emergency purchases	4 times and below	21 (47.73)
	5–7 times	18 (40.91)
	8–12 times	3 (6.78)
	> 12 times	2 (4.58)

### Findings of the qualitative assessment

To complement the quantitative result, in-depth interviews were conducted with CEOs, medical directors, PHCU heads, department heads, and procurement department regarding major challenges faced procurement management performance in the health facilities. A total of 14 key informants (6 from hospitals and 8 from health centers) were involved. The key informants raised the challenges of maintaining good pharmaceutical procurement performance generally from internal and external aspects. External challenges were unavailability of pharmaceuticals, limitations of the procurement system supplier uncertainty, and shortage of hard currency. The internal challenges were lack of failure to make plans in a timely manner, budget shortages and financing-related issues, workforce-related issues, and transportation problems.

### External challenges

The unavailability of pharmaceuticals was mentioned to be a big challenge in ensuring a reasonable level of pharmaceutical procurement performance. One of the key informants described this as:

*“Almost a month for the stock out of laboratory reagents and chemicals; we stopped giving CBC, chemistry and electrolytes such as T3 & T4 diagnostic services because no other option than this sole agent”* [Male, Pharmacy Department head from Hospital]

According to key informants, pharmaceutical’s procurement is within the large national legal framework of the procurement system. Limitations of the procurement system were said to create challenges to ensuring good procurement performance. One of the key informants stated as follows:

*“The procurement system isn’t comprehensive and flexible enough for a health service facility which is life-saving.”* [Male, CEO from hospital]

Interviewees tried to mention the process that health facilities undergo to purchase pharmaceuticals from private suppliers after obtaining a stock out certificate from EPSA. Majority of key informants argued that there were uncertainties from both public and private supplies. Regarding suppliers, one of the participants reported as follows:

*“About 50 % of pharmaceuticals are obtained from EPSA after long process, averagely for 2 weeks. The government has to closely support and supervise EPSA or establish at least two public suppliers to improve pharmaceuticals availability” [Male, Pharmacist from Hospital]”*

Interviewees also revealed that problems related to shortage of hard currency, impede procurement of pharmaceuticals according to plan. One of the key informants said:

*“Sole suppliers did not provide sufficient laboratory commodities as terms and conditions of the contract.”* [Male, pharmacy service, from hospital]

### Internal challenges

According to the key informants, public institutions shall be required to prepare a procurement plan supported by action plans enabling them to execute in due time. With regard to procurement plan and quantification, the majority of interviewees claimed failure to make plans in a timely manner was a major challenge. One of the key informants reported as follows;

*“Due to lack of commitment, lack of functional DTC, and facility-specific medicine list we couldn’t plan timely. Requests were raised from wards, laboratory or other services rather than from pharmacy stores”*

Budget shortages and financing-related issues were also mentioned by the participants. The interviewees described that the allocated budget was not based on actual consumption or match service level of the facility. As a result, public health facilities were unable to acquire the needed pharmaceuticals timely in the budget year. One of the key informants reported:

*“The hospital provides services for a minimum of 800 beds patients, and the allocated budget is insufficient to procure the needed pharmaceuticals for all cases.”* [Male, CEO from Hospital]

Key informants were asked about procurement staff number, qualification, skills and training. Interviewees from health centers and primary hospitals tried to address the several challenges faced concerning the pharmacy workforce. They complained that there was high attrition rate of pharmacy manpower. Among health centers, getting experienced and skillful pharmacists or druggists is difficult; instead, they use clinical nurses. One of the interviewees reported the following:

*“Primary hospital has many constraints; there is high attrition rate of pharmacy manpower; procurement staffs lack experience, skills, and commitment to achieve good pharmaceutical procurement performance.”* [Male, medical director from hospital]

*“We don’t have pharmacy professionals, instead we assigned experienced clinical nurses for pharmaceutical procurement management.”* [Male, PHCU director]

Transportation problem was also investigated and most of the participants explained that health facilities lack transportation to move procured pharmaceuticals. They mentioned that the cost of transport is high at times corresponding to about half of the cost of the products. When mentioning transport problems, one of the key informants explained as:

*“Transport costs are another critical challenge for the poor procurement performance; we rent for 4,000.00 ETB per single load trip of private vehicle from Omonada to Jimma city.”* [Male, Finance head from HC]

## Discussion

This study revealed that health professionals’ perception of transparency, accountability, and efficiency in pharmaceutical procurement performance was low. Lack of efficiency, accountability and transparency in public procurement negatively affects the whole process and systems and leads to mismanagement of public resources [32].

Accountability can improve the performance of the health system by controlling corruption, assuring compliance with standards and procedures. The study sought to find the level of agreement of the respondents on the accountability of pharmaceutical procurement management in public health facilities. The results indicated that pharmaceutical procurement was scrutinized by internal audit. According to the survey findings, only 54% and 57.1% of the health centers and hospitals, respectively, had their own EML indicating low accountability in the selection of the cost-effective of pharmaceuticals. This study was inconsistent with the study conducted in Addis Ababa, Ethiopia on the assessment of the pharmaceutical logistics system in which 93.5% of health centers had their own EML [30]. The difference might be due to high functional DTC (98%) in the reference study. The findings from survey analysis revealed that only 5.4% health centers had a procurement committee and 73% of HCs had functional DTC indicating a low level of separated responsibility for procurement functions among HCs. About 71% of the hospitals had functional DTC. This study result is lower than the survey conducted by the national status assessment of functional DTC in which about 93% in hospitals [37].

Increasing competition among suppliers and products usually decreases pharmaceutical prices. From the study results, only a single hospital (Jimma University Medical Center) used an open tender method as it is the only large Hospital serving more than 15 million catchment populations in the area. More than half, (59.5%) of the participants agreed that the facilities evaluate suppliers after submission of bids and uses generic names for fair competition. The results indicated that health facilities executed fair competition when procuring by the restricted method. The result also implies procurement by generic names encourages generic prescribing principles especially in hospitals. However, 78 (41.1%) of the respondents disagreed that the health facilities evaluate supplier’s qualification based on service reliability, financial capacity, and cost. Their disagreement may be due to the high frequency of emergency purchases, on average five to seven times per year, by a direct method. This study result is in contrast to the study conducted in Kenya, in which all the respondents (100%) agreed that hospitals consider the financial status and on the bases of services reliability [39].

From document review, majority (47.73%) of the health facilities procured up to four times by a direct emergency in 2018/2019 indicating high prices. Fragmented procurement results in high price and wastage of resources causing inefficient procurement management performance. Public health facilities had low practices of supplier performance evaluation, and provision of the right pharmaceuticals at the right time, right quality and right quantity. This outcome further causes budget shortages and more stock-outs, as the result highly impacts service delivery. The study findings are much lower than the study conducted in public hospitals in Ethiopia [24]. The difference might be due to the limitation of the reference study for the exclusion of health centers.

The findings revealed that hospitals procured frequently from EPSA. But the qualitative study result showed that health facilities obtained only up to 50% of their requirements from EPSA after lengthy process averagely for 2 weeks. Thus, competition is an essential factor in achieving this objective and promotes efficiency and effectiveness in procurement, discourages monopoly situations and avoids favoritism. The interviewees also elaborated that besides the higher costs, EPSA did not supply the required pharmaceuticals that resulted in interruption of services provision. These outcomes further affected the inventory management of the health facilities. This study result is argued by a study from Malawi`s public health care delivery findings (42).

The qualitative study also elaborated that HCs face workforce shortages for managing procurement. Personnel who manage procurement are often selected from DTC by management committee indicating low separation of responsibility for selection and procurement, which showed low transparent procurement process. An open and transparent procurement process increases efficiency, reduces the possibility of unfairness or corruption and improves competition [32, 38].

Even though this study has much strength, it has a few limitations. The involvement of pharmaceutical procurement staff in the study might introduce bias. However, to limit the bias, the key indicators were coded by ‘scales’ and assessed using sub-indicators. Moreover, retrospective procurement document review and assessment with the checklist were also conducted. Besides, the absence of similar published quantitative research articles conducted in public pharmaceutical procurement performance with respect to the basic principles (accountability, transparency, competitiveness, and value for money) and similar setup, limits benchmark of the study. Moreover, the study was a public health facility-based cross-sectional study that limits the findings to generalize to the private, military, NGOs health facilities and other actors in the pharmaceutical procurement and supply chain process.

## Conclusion

The study revealed that health professionals’ perception on the level of competitiveness in public pharmaceutical procurement was high but low in terms of transparency, accountability and efficiency that could lead to mismanagement of public resources. Moreover, there is a need to give attention for the monitoring and evaluation of procurement management performance of the health facilities with key indicators regarding transparency, accountability and efficiency. More elaborated studies across different pharmaceutical procurement institutions such as privates, public suppliers, wholesalers, importers and others are also crucial.

## Data Availability

All data generated or analyzed during this study are included in this article.

## Abbreviations

AOR: Adjusted odds ratio
CI: Confidence interval
COR: Crude odds ratio
EML: Essential medicines list
EPSA: Ethiopian Pharmaceutical Supply Agency
FMOH: Federal Ministry of Health
LIAT: Logistics indicators assessment tool
SPSS: Statistical Package for Social Sciences
WHO: World Health Organization
